# Postlecture Evaluation of a Positive Youth Development Subject for University Students in Hong Kong

**DOI:** 10.1100/2012/934679

**Published:** 2012-07-31

**Authors:** Daniel T. L. Shek

**Affiliations:** ^1^Department of Applied Social Sciences, The Hong Kong Polytechnic University, Hong Kong; ^2^Public Policy Research Institute, The Hong Kong Polytechnic University, Hong Kong; ^3^Department of Social Work, East China Normal University, Shanghai 200062, China; ^4^Kiang Wu Nursing College of Macau, Macau; ^5^Division of Adolescent Medicine, Department of Pediatrics, Kentucky Children's Hospital, University of Kentucky College of Medicine, Lexington, KY 40506, USA

## Abstract

The purpose of this study was to examine the postlecture evaluation by the students taking a course (Tomorrow's Leaders) that attempted to promote their leadership qualities and intrapersonal competencies at The Hong Kong Polytechnic University in Hong Kong. Except for the last lecture, students were invited to respond to a 12-item postlecture questionnaire after each lecture. Results showed that the students had positive perceptions of the subject, class, and teacher attributes, and they had positive global evaluation of the teacher and the subject. The postlecture evaluation questionnaire was found to possess good psychometric properties. Multiple regression analyses showed that subject, class, and teacher attributes were predictive of global evaluation of the lecture and the teacher. In conjunction with other evaluation findings, the present findings strongly suggest that students had positive perceptions of the attributes and benefits of “Tomorrow's Leaders.”

## 1. Introduction

Client satisfaction or subjective outcome evaluation is a widely used evaluation method in human services. The common form of subjective outcome evaluation is to distribute a client feedback questionnaire to the clients which may have both quantitative rating items and open-ended questions. In the social welfare context, social workers normally invite the program participants to complete a client satisfaction questionnaire at the end of the program. For example, in the Project P.A.T.H.S. in Hong Kong, subjective outcome evaluation is used to capture the views of the program participants as well as the program implementers. For the program participants, a subjective outcome evaluation form (Form A) is used to gauge their perceptions of the program, instructors, and effectiveness of the program. On the other hand, subjective outcome evaluation forms (Form B and Form C) are used to assess the perceptions of the program implementers on the program, implementers, and effectiveness of the program. Previous research findings showed the value of subjective outcome evaluation strategies in assessing program effectiveness [1–4].

Subjective outcome evaluation is also commonly used in the education sector. For example, it is a common practice for universities throughout the world to evaluate the feedback of students using subjective outcome evaluation. A review of the literature shows that many measures have been developed and studies have been conducted to examine their psychometric properties in the Western world. For example, Cohen proposed that six dimensions of teaching (skills, rapport, structure, difficulty, interaction, and feedback) could be used to assess student feedback [5]. With reference to the Students' Evaluations of Educational Quality (SEEQ), Marsh and Roche [6] identified nine dimensions of student feedback, including learning, teacher enthusiasm, organization, group interaction, individual rapport, breadth of coverage, examinations, assignments/readings, and workload. The SEEQ was translated into Chinese and there was support for the validity of the assessment tool [7, 8]. Kim et al. [9] identified eight broad dimensions underlying course evaluation, including teacher character traits, management of the class, assignments, course design, testing, grading, feedback, and course materials. Kember et al. [10] used the Student Feedback Questionnaire to evaluate teaching which included six dimensions. These dimensions were learning outcomes, interaction, individual help, organization and presentation, motivation, and feedback. 

Several observations can be highlighted from the literature review on course evaluation based on subjective outcome evaluation method. First, while different conceptual frameworks were adopted in different studies, there were similarities across studies. For example, many researchers proposed qualities of the teacher, objectives, teaching techniques, and teacher-student relationship as the basic dimensions of evaluation in their frameworks. Second, exploratory and confirmatory factor analyses were commonly used to examine the underlying dimensions of different course evaluation instruments. Nevertheless, while factor analytic might yield findings that can support elegant statistical models, interpretation of the findings is not always simple. For example, with reference to the framework proposed by Kember and Leung [11], although the proposed model provided an adequate fit to the data, items in the “Challenging Beliefs” and “Motivation” domains are not conceptually pure. Third, in contrast to the vast number of related studies in the West, there are very few studies on Chinese course evaluation questionnaires in different Chinese contexts. Fourth, different course evaluation questionnaires are used by different tertiary institutions in Hong Kong with different dimensions covered in the evaluation questionnaires. In fact, different institutions differ widely on the design of course evaluation questionnaires. Besides, psychometric properties of the instruments are rarely reported. Published scientific findings on the reliability, validity, and norms of the course evaluation assessment tools are almost nonexistent. As such, there is a need to document the psychometric properties of course evaluation tools. Finally, although there are questionnaires on course evaluation, effort to evaluate individual lecture (i.e., postlecture evaluation) is comparatively weak.

While postcourse evaluation can give a global picture about the quality of the course and teacher performance, it is argued that evaluation of individual lectures (i.e., postlecture evaluation) is equally important for several reasons. First, postlecture evaluation can give detailed information about the relevance of the lecture content and quality of lecture delivery. Such specific information is helpful for lecture improvement. Second, as postcourse evaluation takes place at the end of a course, its timeliness in feedback is not quick. In contrast, postlecture evaluation can yield immediate information that can be used by the instructor to plan for the next lecture. Finally, it can be argued that postcourse evaluation may have greater bias because students respond according to their general impression only. Besides, memory decay and reconstruction may affect the recalled information. On the other hand, postlecture evaluation enjoys the advantage of immediacy where students can evaluate the lecture based on the information freshly acquired. Against this background, the present study attempted to carry out postlecture evaluation of a course on positive youth development for university students in Hong Kong.

Under the new 4-year curriculum in The Hong Kong Polytechnic University, there are 30 credits in the General University Requirements (GUR) as follows: (a) Language and Communication (9 credits); (b) Freshman Seminar (3 credits); (c) Leadership and Intrapersonal Development (3 credits); (d) Service Learning (3 credits); (e) Broadening Subjects chosen from 4 clusters (12 credits); (f) Healthy Life Style (Non-Credit Bearing). With specific reference to the requirement in Leadership and Intrapersonal Development, a subject entitled “Tomorrow's Leaders” was developed by the author based on the positive youth development framework. The positive youth development constructs covered in the course included self-understanding, emotional competence, cognitive competence, resilience, spirituality, social competence, moral competence, positive identity, interpersonal communication, conflict resolution, relationship building, and assertiveness. Through lectures, class activities, and assignments, students are helped to understand the attributes of a successful leader, conduct personal reflections, and cultivate their awareness of the importance of intrapersonal and interpersonal attributes of university students (see the appendix). Conceptually speaking, the topics covered in the course are based on the positive youth development framework which is also adopted in the Project P.A.T.H.S. in Hong Kong, with HK$400 million for the initial phase and HK$350 million for the extension phase. To date, evaluation findings based on different evaluation strategies showed two observations: (a) different stakeholders generally had positive views of the program, implementers, and effectiveness and such perceptions were consistent across different stakeholders; (b) compared with control participants, students in the experimental schools had better positive development and they displayed lower levels of substance abuse, delinquency behavior, and intention to engage in risk behavior [12–16]. 

According to the subject syllabus, the objectives of the course are (a) to enable students to learn and integrate theories, research and concepts of the basic personal qualities (particularly intrapersonal and interpersonal qualities) of effective leaders; (b) to train students to develop and reflect on their intrapersonal and interpersonal qualities; (c) to promote the development of an active pursuit of knowledge on personal qualities in leadership amongst students. On successfully completing this subject, it is expected that students will be able to (a) understand and integrate theories, research and concepts on the basic qualities (particularly intrapersonal and interpersonal qualities) of effective leaders in the Chinese context; (b) develop self-awareness and understanding of oneself; (c) acquire interpersonal skills; (d) develop self-reflection skills in their learning; (e) recognize the importance of active pursuit of knowledge on intrapersonal and interpersonal leadership qualities.

The proposed subject was piloted in the second term of 2010/11 school year. To understand the effectiveness of the course, multiple evaluation strategies were used. First, objective outcome evaluation utilizing a one-group pretest-posttest design was used where pretest and posttest data were collected from the students taking the course. Second, postcourse subjective outcome evaluation was conducted where students were invited to respond to a subjective outcome evaluation form including items assessing their perceptions of the course, instructor, and perceived effectiveness of the program at the last lecture. Third, process evaluation via systematic observations was carried out by two trained colleagues to understand the program implementation details in 14 lectures as well as program adherence. Fourth, qualitative evaluation via focus groups involving students based on schools randomly selected from the participating schools was carried out. Finally, qualitative evaluation using reflection notes was conducted. In this paper, findings based on the quantitative data collected in postlecture evaluation are reported in this paper.

## 2. Method

### 2.1. Participants and Procedures

The subject was offered to four classes of students, with a total of 268 students (65 in Class A, 68 in Class B, 66 in Class C, and 69 in Class D). At the end of Lecture 1 to Lecture 13, students were invited to respond to a subjective outcome evaluation form on their perceptions of the content of the lecture and their views. There are 12 items and one open-ended question in the evaluation form. The items cover various areas of lecture, including design, atmosphere, peer interaction, student interest, student participation, opportunities for reflection, degree of helpfulness to personal development, instructor's mastery of lecture, instructor's use of teaching methods, helpful of the lecture to students, global evaluation of lecture, and global evaluation of the lecturer. The respondents were required to respond on a six-point scale with “Strongly Disagree,” “Disagree,” “Slightly Disagree,” “Slightly Agree,” “Agree,” and “Strongly Agree” as the response options. The items are as follows.

- Item 1: The design of this lecture was very good.
- Item 2: The classroom atmosphere of this lecture was very pleasant.
- Item 3: There was much peer interaction amongst the students in this lecture.
- Item 4: I am interested in the content of this lecture.
- Item 5: There was much student participation in this lecture.
- Item 6: There were many opportunities for reflection in this lecture.
- Item 7: This lecture is helpful to my personal development.
- Item 8: The lecturer had a good mastery of the lecture material.
- Item 9: The lecturer used different methods to encourage students to learn.
- Item 10: The lecturer in this lecture was able to help students understand the knowledge covered in the lecture.
- Item 11: Overall speaking, I have very positive evaluation of the lecturer in this lecture.
- Item 12: Overall speaking, I have very positive evaluation of this lecture.

Conceptually speaking, it was hypothesized that items 1, 4, 6 and 7 are related to the attributes of the subject (Subject Attributes), items 2, 3 and 5 are related to the attributes of the class (Class Attributes), and items 8, 9 and 10 were related to the attributes of the teacher (Teacher Attributes). Item 11 and Item 12 are items that are designed to assess the global evaluation of the teacher and the course.

A total of 2,039 questionnaires were collected for all lectures throughout the course. On the day of data collection, the purpose of the evaluation was mentioned, and confidentiality of the data was repeatedly emphasized to all students. All participants responded to the items and question in the evaluation form in a self-administration format. Adequate time was provided for the participants to complete the questionnaire. In the present paper, focus would be put on the findings based on the quantitative data.

### 2.2. Data Analysis

Percentage analyses were used to examine the perceptions of the students on the course and teacher performance. Factor analysis was performed for the Lecture 1 data (i.e., first batch of data) to examine the structure of Item 1 to Item 10 to see whether there was supported for the three dimensions—subject attributes, class attributes and teacher attributes. To examine whether subject attributes, class attributes and teacher attributes predicted the overall evaluation of the teacher (Item 11) and the subject (Item 12), multiple regression analyses were performed. All analyses were performed by using the Statistical Package for Social Sciences Version 16.0.

## 3. Results

A total of 2,039 postlecture subjective outcome evaluation forms were collected after Lecture 1 to Lecture 13. The quantitative findings based on the closed-ended questions are presented in this paper, with the percentage findings presented in Table 1 and the mean findings presented in Table 2. Several observations can be highlighted from the percentage findings presented in Table 1. In the first place, most participants generally had positive perceptions of the course, including its design (Item 1), student interest (Item 4), reflection (Item 6), and benefits (Item 7). For example, 91% of the participants regarded the program design as positive; 85% of the participants agreed that class promoted reflection. Besides, students perceived the class atmosphere to be pleasant (Item 2: 87%), with much peer interaction (Item 3: 88%) and student participation (Item 5: 86%). Finally, teachers were perceived to have good mastery of the course (Item 8), used varied teaching methods (Item 9), and were able to help students understand knowledge (Item 10). Regarding global evaluation of the subject, 93% and 90% had positive evaluation of the teacher and subject, respectively. 

Concerning the psychometric properties of the scale, reliability analysis showed that the 12-item scale was internally consistent in different lectures (Table 1). Both alpha and mean interitem correlation coefficients were found to be in the high range. Regarding the factor structure of the 10 specific items, principal factor analysis followed by promax rotation showed that three factors could be meaningfully extracted, accounted for 75% of the variance. Factor I included items 1, 4, 6, and 7, and it was labeled a Subject Attributes factor (alpha = .85, mean interitem correlation = .59). The second factor included items 2, 3, and 5 (alpha = .85, mean interitem correlation = .66). Because these items are basically concerned with lecture delivery in class, this factor was labeled Class Attributes factor. The third factor included items 8, 9, and 10 that could be labeled a Teacher Attributes factor (alpha = .83; mean interitem correlation = .61). The pattern matrix can be seen in Table 3.

To examine how the subject, class, and teacher attributes contributed to the global evaluation of the teacher and the course, multiple regression analyses were carried out for the data collected from each lecture. Multiple regression analyses showed that subject, class, and teacher attributes predicted global evaluation of the teacher and lecture (Table 4). Among these different aspects, findings showed that subject and teacher attributes showed greater influence on the global evaluation of the teacher and the class.

## 4. Discussion

The present paper examines the postlecture subjective outcome evaluation of a subject entitled “Tomorrow's Leaders” offered at The Hong Kong Polytechnic University. Several observations can be highlighted from the present study. First, the students generally perceived the subject positively in terms of the subject, class, and teacher attributes. The findings also showed that very high proportions of the students had positive global evaluation of the teacher and the subject. Consistent with other forms of evaluation, the present findings showed that the students had positive evaluation of the subject.

Concerning the psychometric properties of the 12-item postlecture subjective outcome evaluation form, reliability analyses showed that the scale was highly reliable in different lectures. Furthermore, consistent with the original conceptual model, factor analyses showed that three dimensions, including subject attributes, class attributes, and teacher attributes were identified and reliability of the related subscales were on the high side. As there are every few published studies on postlecture evaluation in different Chinese contexts, the present findings are interesting additions to the literature. As client satisfaction surveys are commonly criticized as invalid in the field of human service, there is a need to develop validated measures in this field. As pointed out by Royse [17], using validated measures of client satisfaction would “eliminate many of the problems found in hastily designed questionnaires” (page 265). Hence, the present study is a positive response. Nevertheless, it is noteworthy that there are several limitations of the present study. First, as the present findings were based on small samples, there is a need to replicate the findings in large samples. Second, future studies should examine the validity of the 12-item postlecture subjective outcome evaluation form. Third, as the sample size was small, the stability of the factors should be examined in future studies.

Finally, regarding predictors of perceived effectiveness of the course based on the data in different lectures, findings showed that subject, class, and teacher attributes predicted global evaluation of the teacher, although subject and teacher attributes appeared to be stronger predictors. Similarly, although there are findings showing that subject, class, and teacher attributes predicted global evaluation of the subject, subject and teacher factors were stronger predictors. These findings concur with the previous findings that program and implementer characteristics are important factors leading to program effectiveness [18]. With specific reference to program implementers, Donnermeyer and Wurschmidt [19] asserted that implementers' “level of enthusiasm and support for a prevention curriculum influences their effectiveness because their attitudes are communicated both explicitly and subtly to students during the time it is taught and throughout the remainder of the school day” (page 259-260). However, it is noteworthy that there are few studies of predictors of effectiveness of intervention programs. Berkel et al. [20] remarked that “program evaluations have rarely examined more than one dimension in a single study and thus have not untangled possible relations between them” (page 24). Durlak and DuPre [21] further argued that most of the intervention studies failed to examine the relative importance of different predictors of program effectiveness. Hence, the present study is a constructive response to these criticisms.

Methodologically speaking, there may be queries on the use of multiple regression in looking at the relationships between the specific aspects and the global outcomes because it is expected that subject, student, and class attributes are highly correlated. However, it is noteworthy that multiple regression analysis is frequently used to examine predictors of program effectiveness. For example, Byrnes et al. [22] showed that program adherence and quality of program implementation were significant predictors of participants' satisfaction towards the program. Of course, the use of structural equation modeling would give a clear picture on the factors affecting program effectiveness in future.

There are several limitations of this study. First, as only four classes of students were involved in this study, it would be desirable to include more students so that the generalizability of the findings could be enhanced. In addition, it would be helpful to examine the postlecture evaluation findings in different groups of students. For example, it would be interesting to ask whether the subject has different impact for social science and nonsocial science students. Second, the limitations of using a quantitative approach to examine the subjective experiences of the informants should be noted. The use of qualitative techniques in this context would be very helpful. In the present study, data based on one open-ended question were collected and the findings would be reported in another study. Third, as there are many threats to the internal validity of a one-group pretest and posttest research design, addition of a control group can help to examine the impact of the intervention on the program participants. Despite these limitations, the present study is a ground-breaking study in different Chinese contexts and it is a good response to the appeal that psychosocial competencies should be promoted in university students [23].

## Figures and Tables

**Table 1:** Percentage findings based on subjective outcome evaluation of each lecture.

	Percentage of positive responses for different lectures													
Item	1	2	3	4	5	6	7	8	9	10	11	12	13	Overall
(1) Good lecture design	95.6	91.4	83.7	87.1	90.0	93.7	93.8	91.4	93.5	95.2	93.9	87.3	84.2	91.0
(2) Atmosphere was very good	94.3	95.0	80.1	80.6	77.3	84.1	86.0	92.6	89.9	90.3	82.4	83.9	80.9	86.7
(3) Much peer interaction	94.3	95.0	83.2	91.6	80.7	84.9	89.9	93.2	89.9	91.6	85.0	76.7	76.8	88.1
(4) Interested in the content	88.5	88.2	84.7	76.8	87.2	92.1	89.1	93.2	86.9	92.4	85.8	87.3	83.2	87.4
(5) Much student participation	93.8	95.9	85.1	84.5	76.6	82.5	86.8	86.3	91.1	91.7	81.1	75.4	74.7	86.3
(6) Many opportunities for reflection	81.4	89.6	82.7	81.7	86.4	92.9	88.4	85.7	91.1	87.6	81.6	77.1	72.3	84.9
(7) Helpful to my personal development	89.9	90.5	88.6	84.5	89.4	95.2	86.0	88.3	89.3	88.1	88.5	89.0	78.9	88.5
(8) Lecturer had good mastery of lecture	95.2	94.1	93.1	89.7	90.8	96.0	93.8	91.4	93.4	96.6	89.9	86.3	86.3	92.4
(9) Varied teaching methods used	93.0	96.8	89.6	92.9	84.4	87.2	90.6	90.1	92.3	90.3	91.2	84.7	86.3	90.6
(10) Helpful to students (knowledge)	94.7	92.3	93.1	89.7	90.8	95.2	94.5	91.4	92.9	93.1	91.2	89.8	81.1	91.9
(11) Very positive evaluation of the lecturer	95.6	95.0	91.1	92.9	90.8	94.4	96.9	95.1	94.0	92.4	92.6	87.3	83.2	92.9
(12) Very positive evaluation of the lecture	93.0	93.2	86.6	87.7	89.4	92.9	88.4	94.4	92.8	93.1	89.2	89.0	80.0	90.4
Coefficient alpha for the 12-item scale	.93	.93	.94	.95	.95	.95	.94	.93	.94	.94	.94	.95	.97	.94
Mean interitem correlation	.54	.52	.57	.59	.60	.62	.57	.53	.59	.57	.57	.63	.71	.58
Number of questionnaires collected	**227**	**222**	**202**	**155**	**141**	**126**	**129**	**162**	**169**	**145**	**148**	**118**	**95**	**2039**

Note: The cumulative percentage based on “Strongly Agree”, “Agree” and “Slightly Agree” for an item is presented for each lecture.

**Table 2:** Subjective outcome evaluation of each lecture.

	Lecture													
Item	1	2	3	4	5	6	7	8	9	10	11	12	13	Mean
(1) Good lecture design	4.59	4.56	4.31	4.36	4.55	4.63	4.52	4.50	4.58	4.54	4.49	4.33	4.23	4.48
(2) Atmosphere was very good	4.77	4.75	4.26	4.26	4.26	4.37	4.43	4.59	4.53	4.50	4.36	4.23	4.18	4.42
(3) Much peer interaction	4.86	4.92	4.36	4.48	4.34	4.34	4.57	4.70	4.54	4.52	4.40	4.16	4.09	4.48
(4) Interested in the content	4.41	4.48	4.37	4.24	4.43	4.60	4.47	4.54	4.53	4.53	4.41	4.31	4.34	4.43
(5) Much student participation	4.71	4.75	4.37	4.32	4.18	4.23	4.44	4.55	4.61	4.62	4.30	4.07	4.12	4.41
(6) Many opportunities for reflection	4.19	4.37	4.22	4.37	4.54	4.68	4.53	4.35	4.60	4.37	4.29	4.21	4.01	4.36
(7) Helpful to my personal development	4.39	4.44	4.36	4.32	4.50	4.72	4.41	4.42	4.53	4.43	4.44	4.38	4.19	4.42
(8) Lecturer had good mastery of lecture	4.73	4.72	4.52	4.52	4.61	4.69	4.78	4.57	4.67	4.68	4.61	4.48	4.37	4.61
(9) Varied teaching methods used	4.79	4.78	4.45	4.53	4.50	4.54	4.64	4.52	4.65	4.65	4.56	4.34	4.31	4.56
(10) Helpful to students (knowledge)	4.52	4.58	4.41	4.43	4.52	4.62	4.62	4.45	4.58	4.51	4.52	4.41	4.14	4.49
(11) Very positive evaluation of the lecturer	4.72	4.75	4.43	4.51	4.57	4.71	4.71	4.53	4.66	4.57	4.52	4.38	4.31	4.57
(12) Very positive evaluation of the lecture	4.59	4.64	4.32	4.37	4.45	4.63	4.56	4.53	4.60	4.51	4.41	4.37	4.23	4.48
Number of questionnaires collected	**227**	**222**	**202**	**155**	**141**	**126**	**129**	**162**	**169**	**145**	**148**	**118**	**95**	**2039**

**Table 3:** Pattern matrix for the 10 specific items on different aspects of the lecture.

Item	Subject Attributes	Class Attributes	Teacher Attributes
(1) Good lecture design	**.426**	.377	.080
(2) Atmosphere was very good	−.023	**.810**	.092
(3) Much peer interaction	−.104	**.918**	−.050
(4) Interested in the content	**.661**	.172	.015
(5) Much student participation	.163	**.567**	.125
(6) Many opportunities for reflection	**.795**	−.180	.111
(7) Helpful to my personal development	**.865**	.011	−.120
(8) Lecturer had good mastery of lecture	.151	.005	**.650**
(9) Varied teaching methods used	−.170	.098	**.866**
(10) Helpful to students (knowledge)	.187	−.030	**.663**
Variance Explained (%)	53.4	6.49	4.15

**Table 4:** Predictors of global evaluation of the teacher and global evaluation of the lecture.

Analyses	Lecture													
	1	2	3	4	5	6	7	8	9	10	11	12	13	Overall
DV: Global perception of the teacher (Item 11)														

*Predictors*														

Subject attribute	.32***	.28***	.53***	.34***	.36***	.56***	.32**	.16	.26**	.00	.40***	.25*	.21	.31***
Class attribute	.06	.21**	.10	.07	.00	.15	.06	.26**	.06	.30***	.08	.18	.21	.12***
Teacher attribute	.49***	.39***	.27***	.47***	.56***	.43***	.47***	.32**	.54***	.59***	.34***	.44***	.54***	.45***
*R* square	.61	.59	.68	.65	.76	.66	.58	.45	.63	.65	.54	.65	.82	.63

DV: Global Perception of the Course (Item 12)														

*Predictors*														

Subject attribute	.39***	.48***	.57***	.45***	.39***	.53***	.21*	.28**	.26**	.43***	.46***	.38**	.34*	.41***
Class attribute	.14*	.07	.22***	.20**	.17*	.03	.42***	.26**	.19*	.24**	.18*	.11	.18	.19***
Teacher attribute	.36***	.36***	.13*	.26***	.33***	.37***	.33***	.28**	.41***	.24**	.27***	.37***	.39***	.30***
*R* square	.63	.66	.71	.67	.66	.68	.71	.56	.63	.67	.68	.65	.76	.66

Number of questionnaires collected	**227**	**222**	**202**	**155**	**141**	**126**	**129**	**162**	**169**	**145**	**148**	**118**	**95**	**2039**

Note: ***P < .001; **P < .01; *P < .05.

**Table 5:** Topics covered in different lectures.

Lecture	Subject Content
1	An overview of leadership theories and exploration of the meaning of “effective leaders” and personal attributes constituting effective leadership. This overview also helps students understand how leadership studies are closely related to their own daily life experience and personal development.
2	Self-understanding: theories and concepts; personality traits; self-concept; self-esteem and personal identity; the role of self-understanding in effective leadership.
3	Emotional competence: awareness and understanding of emotions; emotional quotient (EQ); role of emotional management in effective leadership.
4	Cognitive competence: different types of thinking; higher-order thinking; experiential learning; role of cognitive competence in effective leadership.
5	Resilience: stresses faced by adolescents; life adversities; coping with life stresses; adversity quotient (AQ); role of resilience in effective leadership.
6	Spirituality: meaning in life and adolescent development; spirituality and mental health; role of spirituality in effective leadership.
7	Ethics and morality: moral issues and moral competence; role of ethics; morality and personal integrity in effective leadership.
8	Social competence: basic social competence skills; ability to build up positive human relationship; respecting the views of oneself and others; role of social competence in effective leadership.
9	Positive personal identity: develop healthy identity formation and achievement in youth; role of positive personal identity in effective leadership.
10	Interpersonal communication: theories, concepts, and skills of interpersonal communication; role of communication skills in effective leadership.
11	Interpersonal conflict and assertiveness: theories of interpersonal conflict; conflict resolution skills; assertiveness and non-assertiveness; role of conflict resolution and assertive skills in effective leadership; student presentations (Group 1 and Group 2).
12	Relationship building and maintenance: tactics of building and maintaining relationship; relationship quality and effective leadership; student presentations (Group 3 and Group 4).
13	Team building: tactics and strategies of team building; identifying common goals in a team; maintaining morale and dealing with demoralization; student presentations (Group 5 and Group 6).
14	Wrapping up; summary of the course; evaluation; student presentations (Group 7 and Group 8).

## Appendix

See Table 5.
